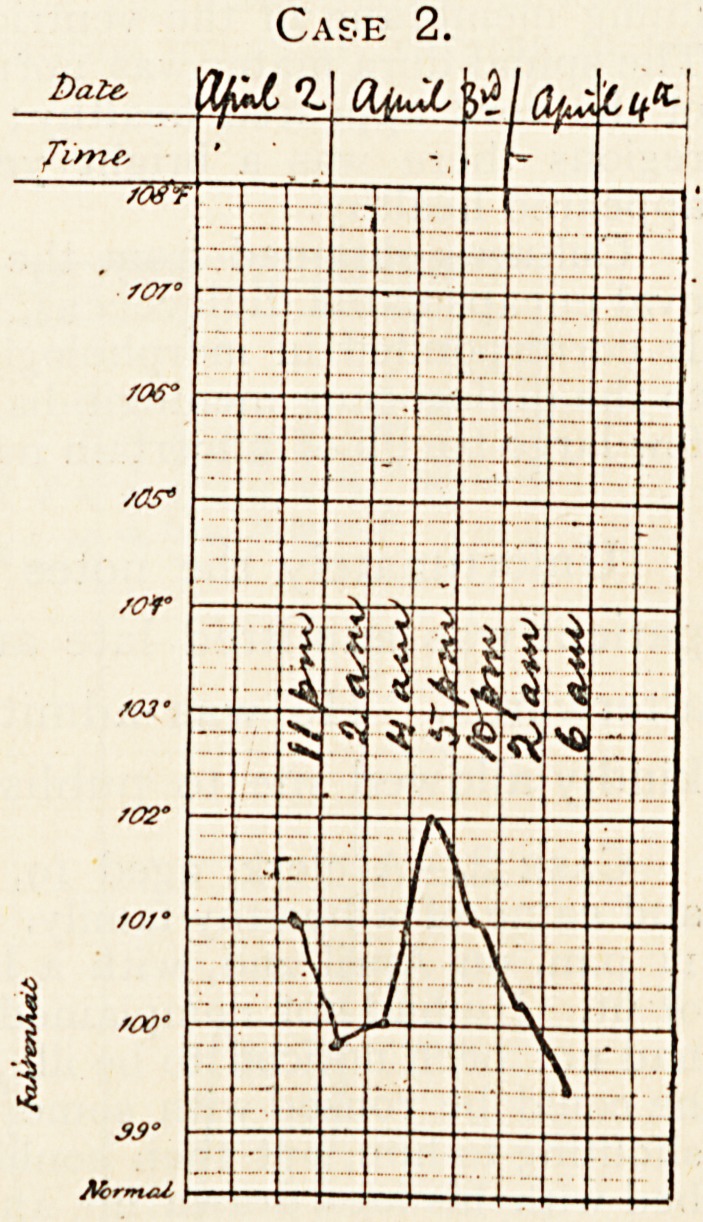# Two Cases of the Sporadic Form of Epidemic Cerebro-Spinal Meningitis

**Published:** 1900-06

**Authors:** J. Michell Clarke

**Affiliations:** Professor of Pathology, University College, Bristol; Physician to the Bristol General Hospital


### TWO CASES OF THE SPORADIC FORM OF EPIDEMIC CEREBRO-SPINAL MENINGITIS.

BY

J. Michell Clarke, M.A., M.D. Cantab., F.R.C.P. Lond.,

*Professor of Pathology, University College, Bristol; Physician to the Bristol General Hospital.*

---

The following two cases of cerebro-spinal fever are the only ones that have occurred at the Bristol General Hospital within the last twelve years, and probably for a much longer period, but of that I have no certain knowledge. It is certainly worthy of remark that both cases were admitted within four weeks. I also learnt that the sister of the first patient was at home ill with high fever and delirium; but as I failed to ascertain the exact nature and result of her illness, I cannot say whether she also suffered from the same disease. According to Jaeger,[1] there is now an epidemic period of the disease in Europe and America. Cerebro-spinal fever is fortunately rare in the epidemic form in this country, the last minor outbreak having taken place in the Eastern Counties in 1890. Sporadic cases, however, occur from time to time. In the United States, where epidemics have been of frequent occurrence and sometimes of great severity, sporadic cases constantly occur in the intermediate years. An interesting point is the relation of the sporadic cases to the epidemic form. In the recent severe epidemic in Boston, U.S.A., 1896–7, the mortality was 68.5 per cent., and in thirty-nine sporadic cases occurring between 1880 and 1896 it was 59 per cent. The symptoms of the disease and the pathological changes are the same in both. The epidemic form of the disease is now generally admitted to be due to the diplococcus intracellularis meningitidis (Weichselbaum); Netter, however, maintains that it may be caused

[1] *Deutsche med. Wchnschr.*, 1899, xxv.

either by this organism or by the pneumococcus.[1] With regard to the sporadic cases, the question seems to be not finally settled, but many observers have also found in these the D. intracellularis meningitidis.

Dr. D. S. Davies kindly undertook the examination of the exudate in the two cases described below. He found in each one a diplococcus corresponding in morphological characters to the D. intracellularis meningitidis, and in the second the pneumococcus was also present. This last is not an infrequent association. Netter found these organisms present together in ten out of thirty-nine cases. I regret that my case was not examined for the presence or absence of Kernig's symptom; but before reading Prof. Osler's Cavendish Lecture in July, 1899, I was not acquainted with this symptom. The account of the cases is as follows:—

Case 1.—Frederick G. S., aged 10, had always been healthy until October 11th, when he was run over, the right side of the jaw and the right collar-bone being fractured. He was brought to the Bristol General Hospital, and remained there until December 11th. On his discharge he was deaf in the right ear, and had a squint, which subsequently disappeared. He was also very irritable. He appeared to have recovered his health until on March 7th he lost his appetite, and on the 9th complained of feeling ill. On the 10th he vomited whilst eating his dinner; the vomiting recurred at frequent intervals during that evening and night. At midday on the 11th he suddenly became feverish and delirious, shouting loudly, and his mother said that his mouth and eyes were alternately turned to the right and left, and the squint returned. At the same time he threw his arms and legs about, throwing his arms over his head and twisting himself from right to left. He passed no urine all this day until the evening, when he passed it in bed. The bowels were constipated. He constantly asked for water; his speech was muffled and unintelligible. At midnight there was a remission of the symptoms until the evening of the 12th, when they returned, and he was admitted on the 13th. Previous to admission, there were no rigors, fits, or paralysis. On examination, he was drowsy, the face flushed, and the corners of the mouth retracted. He was restless and delirious. Pulse, 140, soft; respiration, 24. Tongue covered with a thick yellow fur. Sordes on the teeth and lips. He was badly nourished: the skin was hot and dry. The thoracic and abdominal organs appeared healthy. The abdomen was not retracted; there was no tâche cerebrale. The head was slightly retracted, the muscles at the back of the neck being somewhat rigid. The pupils were not dilated, but the right was larger than the left. The patient lay on his right side, with hips and knees flexed. He strongly objected to being examined, and resisted being disturbed. There was marked photophobia, the eyelids being kept firmly closed: there was right external strabismus. The head and spine were gener-

[1] *See* Osler, Cavendish Lecture, *Brit. M. J.*, 1899, i. 1517.

ally tender to light percussion. On the 14th the patient was delirious and very noisy, constantly shouting out. He lay on his back, with the head very slightly retracted and turned to the right. The pupils were contracted and acted to light. There was some ptosis on the right side. Rigidity of the right shoulder and elbow-joints was observed. The knee-jerks could not be obtained; the superficial reflexes were present. He had not vomited since admission. Examination of the ears showed that they were normal. March 15th.—The patient became quieter during last night, and passed gradually into coma. Herpes labialis had appeared at the left angle of the mouth. His face was pale. The right arm was rigid, the other limbs flaccid. He still lay on his back, with his head turned to the right and partly backwards. The pulse was feeble, 144; the respirations irregular, varying in rate

Case 1.

from 12—16 up to 30—40 in the minute; they were not rhythmical. He lay quiet, but resisted when disturbed, especially if his head was moved. The knee-jerks were absent, the plantar and abdominal reflexes present. The pupils were dilated; there was right ptosis and right external strabismus: no view could be got of the right optic disc, the left was a little reddened, and the veins engorged; its edge was fairly clear. There was decided paresis of the left arm, left leg, and left side of face. During the last two days the bowels had remained obstinately constipated. From the time of admission the urine was passed unconsciously into the bed. He died comatose the same night.

The *post-mortem* examination was made eighteen hours after death. With the exception of labial herpes, there was no eruption on the skin. There were some patches of collapse in the upper lobe of the right lung; the heart cavities were full of pale firm clot. The viscera were otherwise normal. The skull was normal. There was no disease of the middle or internal ears or mastoids. The dura matter was healthy; the longitudinal sinus and the sinuses at the base were full of firm white clot, not adherent to the walls of the vessels. On removing the dura, a bright greenish-yellow exudation covered the whole surface of the cerebrum and cerebellum; it was most abundant over the vertex and over the superior surface of the cerebellum, but extended also over the whole of the base. On section, the substance of the brain was softer than normal, and the grey matter dark in colour. The ventricles were moderately dilated, and contained clear fluid; the lining membrane of the ventricles was soft, like the brain substance. The spinal dura mater was normal, and on removing it the upper part of the cord appeared healthy; but over the lower dorsal and lumbar regions there was a bright yellow exudation. The cranial nerves appeared healthy.

Cultures obtained from the exudation showed the presence of an oval encapsulated diplococcus, which did not stain by Gram's method. It corresponded in morphological characters and staining reactions to the diplococcus meningitidis of Weichselbaum. There were also a few large bacilli of uncertain nature.

Unfortunately the notes of the next case are brief, as the patient was admitted late at night, and died thirty-four hours afterwards. He was admitted under Dr. Harrison, who has kindly allowed me to publish the case.

Case 2.—A clerk, aged 19, who had always enjoyed good health, and came of a healthy family. He was brought into the Hospital at 11 p.m. on April 2nd, with a history of having fallen down in a kind of fit. He had not complained of being out of health previously, and had not been noticed to be ill. On admission he was semi-comatose; he could be roused with some considerable difficulty, by dint of loud shouting to him, but then could not say more than "Ah!" He lay in bed with his legs drawn up, and was very restless, throwing his arms about, and constantly turning from side to side, but lying more on the right side than the left. There was some rigidity of the legs, and he strongly resisted any attempt to straighten them. On account of the violence of his movements, no thorough examination could be made. The head was retracted on the neck, and this retraction gradually increased until his death. The pupils were dilated—there was no squint. There was photophobia. The optic discs could not be examined. There was no discharge from the ears, and, so far as could be seen, no otitis media. The face was symmetrical, and there was an abundant eruption of herpes labialis. There was no paralysis of the limbs. The knee-jerks were not obtained. The urine was passed into the bed. He vomited once a few hours after admission, and was at first unable to swallow, but afterwards took a little milk. On examination, the lungs were normal in front; the heart's apex was in the normal situation, and a systolic murmur was audible at the apex. The abdominal organs were normal. On April 3rd his condition was much the same, but he was much weaker; the pulse was 84, soft; the respirations irregular, 42 to the minute. He was still semi-comatose, and tossing about from side to side. He did not cry out. The retraction of the head had increased. So far as could be made out, there was tenderness to percussion over the head and over the spinal column. The coma gradually deepened during the night, and he died early on the following morning. Some hours before death some purpuric spots appeared on the legs and lower parts of the thighs.

The *post-mortem* examination was made twenty-nine hours after death. The body was well nourished, and rigor mortis was present. The bronchi contained frothy mucus. The lower lobes of the lungs were congested and œdematous, but the lungs were everywhere crepitant. The heart was normal, the liver congested; the spleen was large, dark, and firm, and the Malpighian bodies were prominent. The other abdominal viscera were healthy. The cranium was normal; there was no middle-ear or mastoid disease. The dura mater was healthy. On the pia mater there was a bright yellow purulent exudation, forming a thin layer which completely covered the vertex and convexity of both hemispheres and the cerebellum; it formed a thick layer on the under surface of the cerebellum, but elsewhere over the base the exudation was scanty or absent. The vessels of the pia-mater and of the grey matter of the hemispheres were deeply injected. The cerebral hemispheres appeared to be bulged and the ventricles were filled with clear fluid. The cranial nerves appeared healthy. The spinal dura-mater was healthy. A scanty yellowish purulent exudation similar to that over the brain was found on the pia-mater of the cord, most abundant in the lower dorsal region and on its posterior aspect. The cord itself looked normal, and was not softened. Cultures made on agar-agar from the exudation in this case showed the presence of the pneumococcus, and also of a diplococcus not staining by Gram's method, and corresponding in morphological characters to the diplococcus intracellularis meningitidis. Attempts to grow the latter on ascitic serum failed.

CASE 2.

Microscopic examination was made of sections stained in logwood and eosin, thionin, and methylene blue; for the two latter stains the tissues were hardened in alcohol. The chief changes were found in the brain in the meninges, in which there was marked injection of blood-vessels, and the pia-arachnoid appeared swollen with purulent infiltration. The leucocytes infiltrated the vessel walls, and were especially abundant around them. The largest masses of collections of leucocytes were in the sulci. In the brain itself the superficial vessels of the cortex were greatly distended, with dilatation of the lymph-spaces, which contained leucocytes. The superficial layers of the cortex appeared œdematous; no hemorrhages were found. The nerve-cells of the cortex, for some reason, were not very successfully stained. In the spinal cord, the soft membranes presented the same appearance as those of the brain, but here the greatest mass of leucocytes (purulent foci) was on the posterior surface around the posterior roots, and also on the anterior surface, extending deeply into the anterior fissure. The superficial vessels which supply the circumference of the cord were injected, and this layer of the cord itself stained badly and was perhaps œdematous. Elsewhere the cord was normal, and the cells in all parts of the grey matter stained well and showed a normal structure. Some of the vessels in the meninges contained thrombi. In both brain and cord the relatively large number of leucocytes in the vessels cut in cross-section was very noticeable, and was evidence of a marked leucocytosis. The leucocytes of the exudation on the meninges consisted exclusively of those with a tripartite nucleus (finely granular oxyphil cells), and in many places diplococci could be seen to be included in them.

Pathologically the presence of the diplococcus intracellularis meningitidis in these sporadic cases of the disease is the most interesting feature: in a recent paper Dr. W. J. Buchanan says that it was found in three cases in the Bhagalpur Gaol, India[1]. From the clinical point of view, a marked feature in both cases was the extreme and constant restlessness. I did not see the second case during life, but the sudden onset and rapid course would class it under the fulminating variety of the disease: we had to depend, of course, upon the statements of his friends for the history, but, in any case, it may safely be concluded that there were no symptoms of serious illness until the fit which ushered in the rapidly-fatal attack. The petechial rash on the lower limbs and the labial herpes correspond to two of the skin eruptions most frequently seen in cerebro-spinal fever. In the first patient the sudden onset with high fever, acute delirium, pains in the back and limbs, herpes labialis, slight retraction of head, tenderness over head and spine, photophobia, partial right ptosis, and right external strabismus in the absence of pneumonia and of middle-ear disease, practically the two most frequent causes of a secondary meningitis, made the diagnosis of a sporadic case of cerebro-spinal fever sufficiently clear. For this reason, and also because of the extreme restlessness of the patient, a lumbar puncture was not attempted.

[1] *Indian M. Gaz.* 1899, xxxiv. 436.

## Figures and Tables

**Case 1. f1:**
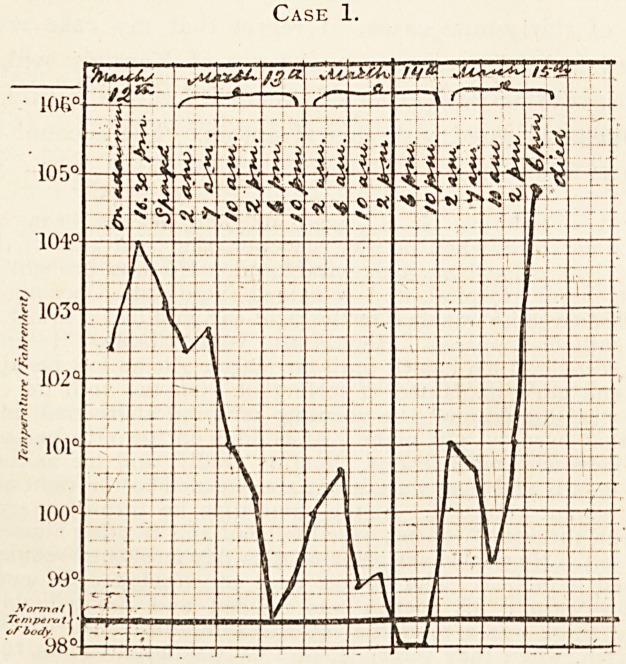


**Case 2. f2:**